# Combination of Dexamethasone and Tofacitinib Reduces Xenogeneic MSC-Induced Immune Responses in a Mouse Model of Alzheimer’s Disease

**DOI:** 10.3390/biomedicines10081882

**Published:** 2022-08-04

**Authors:** Na Kyung Lee, Su Hyeon Myeong, Jung Won Hwang, Jason K. Sa, Hyo Jin Son, Hee Jin Kim, Hyemin Jang, Jong Wook Chang, Duk L. Na

**Affiliations:** 1 School of Medicine, Sungkyunkwan University, Suwon 16419, Korea; 2 Samsung Medical Center, Cell and Gene Therapy Institute (CGTI), Research Institute for Future Medicine, Seoul 06351, Korea; 3 Alzheimer’s Disease Convergence Research Center, Samsung Medical Center, Seoul 06351, Korea; 4 Department of Health Sciences and Technology, Samsung Advanced Institute for Health Sciences & Technology (SAIHST), Sungkyunkwan University, Seoul 06351, Korea; 5 Department of Biomedical Sciences, Korea University College of Medicine, Seoul 02841, Korea; 6 Samsung Medical Center, Department of Neurology, Sungkyunkwan University School of Medicine, Seoul 06351, Korea; 7 Neuroscience Center, Samsung Medical Center, Seoul 06351, Korea; 8 Stem Cell Institute, ENCell Co., Ltd., Seoul 06072, Korea

**Keywords:** immunosuppressant, Alzheimer’s disease, dexamethasone, tofacitinib, immune response

## Abstract

We have recently reported on how transplantation of human mesenchymal stem cells (MSCs) into the mouse parenchyma generated immune responses. To facilitate the clinical translation of MSC-based AD therapy, the safety and efficacy of human derived MSCs (hMSCs) must be confirmed in the pre-clinical stage. Thus, it is imperative to investigate measures to reduce immune responses exerted via xenotransplantation. In this study, immunosuppressants were co-administered to mice that had received injections of hMSCs into the parenchyma. Prior to performing experiments using transgenic AD mice (5xFAD), varying immunosuppressant regimens were tested in wild-type (WT) mice and the combination of dexamethasone and tofacitinib (DexaTofa) revealed to be effective in enhancing the persistence of hMSCs. According to transcriptome sequencing and immunohistochemical analyses, administration of DexaTofa reduced immune responses generated via transplantation of hMSCs in the parenchyma of 5xFAD mice. Significant mitigation of amyloid burden, however, was not noted following transplantation of hMSCs alone or hMSCs with DexaTofa. The efficacy of the immunosuppressant regimen should be tested in multiple AD mouse models to promote its successful application and use in AD stem cell therapy.

## 1. Introduction

Prior to assessing the safety of human mesenchymal stem cells (hMSCs) in clinical trials, the therapeutic efficacy of hMSCs must be assessed in the pre-clinical level and thus performing xenotransplantation is a mandatory step that cannot be evaded. Concerns, though, have been raised regarding the immunogenicity of MSCs [1,2]. We have reported that in contrast to WT mice that received transplantations of allogeneic MSCs, higher immune responses were exhibited from mice transplanted with xenogeneic MSCs into the parenchyma [3]. The use of immunosuppressants alleviated the immune response and increased the persistence of human MSCs in the WT mouse brain [4]. However, whether similar results can be observed in transgenic mouse models have not been investigated.

The overall perspective on the application of MSCs for AD therapy is both favourable and promising [5,6]. Several groups have studied the therapeutic roles of allogeneic or xenogeneic MSCs in transgenic AD mouse models [7]. The MSCs were administered via varying delivery routes such as the intraparenchymal [8], intracerebroventricular (ICV) [9,10], intranasal [11], and intravenous [12] routes. While the ability of MSCs to remove amyloid-beta [12,13,14], enhance proteasomal activity [8], upregulate neurogenesis [10], and improve cognitive performance [15] have been extensively studied, a limited number of studies have investigated the immune responses generated by the transplanted MSCs and how such responses may directly or indirectly affect the overall engraftment of the MSCs in an AD mouse model. Unlike MSCs, due to the immunogenic properties of neural stem cells (NSCs), numerous pre-clinical studies have been conducted where NSC transplantation is combined with administration of immunosuppressants such as tacrolimus [16].

In this study, our major objective was to examine how administering immunosuppressants will affect the immune response and persistence of hMSCs transplanted into the parenchyma of a transgenic Alzheimer’s disease (AD) mouse model. Due to its invasive nature and high cost, the intra-parenchymal route may not be clinically considered as the most ideal route to deliver stem cells. However, the potential cell loss that may arise via injection into the cerebrospinal fluid (CSF) can be circumvented. Thus, the effects of the immunosuppressants on the transplanted MSCs, without having to take into consideration of CSF flow, can be directly examined. Moreover, to increase the translatability of the results, clinically relevant doses and administration routes were used in the study [17,18]. Similar to results previously observed from WT mice [4], we hypothesized that combined therapy involving the use of more than one immunosuppressant will be effective in reducing immune responses generated via hMSCs injected into the parenchyma of the transgenic AD mouse model.

## 2. Materials and Methods

### 2.1. Ethical Statement

This study was reviewed and approved (Approval number: 20200429001; Date: 29 April 2020) by the Institutional Animal Care and Use Committee (IACUC) of the Samsung Biomedical Research Institute (SBRI) at Samsung Medical Center (SMC). The SBRI abides by the Institute of Laboratory Animal Resources (ILAR) guide and is an Association for Assessment and Accreditation of Laboratory Animal Care International (AAALAC International) accredited facility.

### 2.2. Experimental Animals

Mice were housed and bred in cages according to the ILAR guide of SMC. They were fed ad libitum and a 12-h light/12-h dark cycle was maintained in the facility.

C57BL/6 mice that were 6–8-week-old of age were purchased from OrientBio (Gyeonggido, Korea). A total of 61 C57BL/6 mice were used in the study. Out of the 61, 43 mice were used to assess the quantity of residual human MSCs in the mouse parenchyma (hMSC: *n* = 7, hMSC + Dexamethasone (hMSC + Dexa): *n* = 8, hMSC + Tacrolimus (hMSC + Tac): *n* = 6, hMSC + DexaTac: *n* = 7, hMSC + Tofacitinib (hMSC + Tofa): *n* = 9, and hMSC + DexaTofa: *n* = 6). The remaining 18 mice were used to carry out immunohistochemical analysis (MEM: *n* = 2, hMSC: *n* = 3, hMSC + Dexa: *n* = 3, hMSC + Tac: *n* = 3, hMSC + DexaTac: *n* = 3, hMSC + Tofa: *n* = 2, and hMSC + DexaTofa: *n* = 2). For both the hMSC + Tofa and hMSC + DexaTofa groups, originally injections were made in three mice for each group. However, the injection site could not be identified from one out of the three mice, for both groups respectively, and thus histological analysis was performed using two mice for each of the groups.

The transgenic AD mouse model (5x familial Alzheimer’s disease; 5xFAD) was originally purchased from the Jackson Laboratory (Bar Harbor, ME, USA). Genotyping was performed using tail snips from littermates that were born from mating 5xFAD and C57BL6/J mice. Mice that expressed both the amyloid precursor protein (APP) and presenilin 1 (PS1) were transgenic. A total of 26 5xFAD mice that were 9–10 months of age were used in the study. Out of the 26, 6 mice were used to conduct transcriptome sequencing (MEM: *n* = 2 (both males), hMSC: *n* = 2 (both males), and hMSC + DexaTofa: *n* = 2 (both males)). Seven mice were used to quantitate residual hMSCs in the mouse brain (MEM: *n* = 2 (1 male, 1 female), hMSC: *n* = 3 (3 females), and hMSC + DexaTofa: *n* = 2 (1 male, 1 female)) and the remaining 13 mice were used to carry out histological analysis (MEM: *n* = 3 (3 females), hMSC: *n* = 4 (1 male, 3 females), and hMSC + DexaTofa: *n* = 6 (all females)). For ALU qPCR, the MEM group was considered as a negative control and background. If the resulting MSC quantity for each respective mouse was similar or lower than the values acquired for the MEM injected mice, which may have occurred due to the inaccurate injections of hMSCs, those values were considered as background and were excluded from analysis. Originally for the hMSC + DexaTofa group, the MSC quantity for two out of four of the mice was similar or lower than the MEM group and thus were excluded from the final analysis.

### 2.3. Preparation and Administration of Immunosuppressants

Dexamethasone (0190031, 1 mL per ampule; Jeil Pharmaceuticals Co., Seoul, Korea), Tacrolimus (677200101; Astellas Pharmaceuticals, Tokyo, Japan), and Tofacitinib (T-1399-100 mg; LC Laboratories, Woburn, MA, USA) were used in the study (Table 1). All three immunosuppressants were diluted with an isotonic sodium chloride solution (54X1AF3; Dai Han Pharm, Seoul, Korea) prior to administration. The dosages used in the clinical settings for the three immunosuppressants [19,20,21] were referred to calculate the equivalent animal dose [22]. A total of 100 µL of Dexamethasone (Dexa; 2.1 mg/kg) was administered via the intravenous (IV) route on the day of the cell injection (D0). Dexamethasone was injected right before and 4 h after completing the cell injection. Tacrolimus (Tac; 0.6 mg/kg, 100 µL) was administered orally via oral gavage (P.O.) once a day, starting from 2 days before the cell injection (D-2) up to the sacrifice time point (D7). As reported previously [23], Tofacitinib (Tofa) was suspended in 0.5% methylcellulose/0.025% Tween 20 (C5678-500G; Sigma Aldrich, Saint Louis, MO, USA). Tofacitinib was administered orally (1 mg/kg, 100 µL), twice a day from D-2 up to D7.

### 2.4. Intra-Parenchymal Injection of Human MSCs

Previously reported procedures were referred to prepare the cells for injection [4,24]. Human Wharton’s jelly-derived mesenchymal stem cells (Human MSCs; hMSCs) were preconditioned with ethionamide (1261004; Invitrogen, Waltham, MA, USA) and were cultivated in T175 flasks (Thermo Scientific, Waltham, MA, USA) containing MEM alpha 1x (MEMα1x) media (12571-071; Gibco, Waltham, MA, USA) supplemented with 10% foetal bovine serum (FBS, S1480; Biowest, Riverside, MO, USA) and 0.5% Gentamicin (15710-072; Invitrogen, USA) at 37 °C, 5% CO_2_. MSCs were suspended in phenol red free MEMα1x (41061-029; Gibco, USA) prior to injection. Mice were anesthetized with 1.5–2% isoflurane (1015; Hana Pharmaceuticals Co., Seoul, Korea) during the injection. Mice were placed in a prone position and the heads of the mice were firmly fastened using the stereotactic holders (Harvard Apparatus, Cambridge, MA, USA). Upon shaving the hair on the head and disinfecting the skin with povidone-iodine, a skin incision was made. After exposing the skull, the following stereotactic coordinates were used to make a burr hole: A/P −0.5 mm, M/L −1.7 mm, D/V −3.3 mm [3,4]. Using a 25 µL Hamilton syringe, a total of 2 × 10^5^ MSCs suspended in 5 µL of phenol red free MEMα1x was injected into the left caudate putamen (CPu) of each mouse at an injection rate of 1 µL/min. Following a 5 min delay, the syringe was retracted slowly, and the incised area was sutured. The sutured region was again disinfected with povidone-iodine. Mice were allowed to recover in a heating pad before being placed back in their cages. Mice were sacrificed 7 days after the cell injection (D7).

### 2.5. Quantification of Residual Human MSCs in the Mouse Brain

Genomic DNA was extracted (158046; Qiagen, Hilden, Germany) from the left hemisphere where cells were injected (left cerebellum included). The extracted genomic DNA samples, SYBR Green Master Mix probe (4367659; Thermo Fisher Scientific, Waltham, MA, USA), and the ALU primers (Forward: 5′- CAT GGT GAA ACC CCG TCT CTA-3′, Reverse: 5′-GCC TCA GC TCC CGA GTA G-3′) were used to carry out quantitative real-time polymerase chain reaction (qPCR) using the QuantStudio 6 system (Applied Biosystems, Waltham, MA, USA). As ALU is generally used to detect the presence of human cells [4,25,26], it is possible to quantitate residual human MSCs in the mouse parenchyma. The PCR conditions were carried out (total of 40 cycles) by referring to previously reported methods [4]: (1) 95 °C 10 min, (2) 95 °C 15 s, (3) 68 °C 30 s, and (4) 72 °C 30 s. Serial dilutions (10-fold) of gDNA extracted from 10^6^ hMSCs were made to create a standard curve. The cycle threshold (Ct) values of each of the samples were measured and compared to the standard curve to quantitate hMSC persistence in the mouse parenchyma.

### 2.6. Histological Analysis of Fixated Brain Tissue Samples

Harvested brain tissue samples were fixated in 4% paraformaldehyde (PC2031-050-00; Biosesang, Seongnam, Korea) prior to making paraffin blocks. Serial sections (4 µM thick) of the tissue samples were made using a microtome. To perform immunohistochemical (IHC) staining slides were deparaffinized and blocked using 1x citrate buffer (C9999-1000 mL; Sigma, USA). IHC was carried out using previously reported methods [4]. The following primary antibodies were used in this study: anti-CD45 (1:200, 103101; Biolegend, San Diego, CA, USA), amino acid residue 1–16 of beta amyloid Aβ 6E10 (1:250, 803001; Biolegend, USA), STEM121 (1:500, Y40410; Cellartis, San Jose, CA, USA), and PD-1 (1:400, ab214421; Abcam, Waltham, MA, USA). Dako EnVision + System-HRP Labelled Polymer anti-mouse (K4001) and anti-rabbit (K4003) were used (Dako, Carpinteria, CA, USA). A polyclonal goat anti-rat IgG HRP polymer (ab214882; abcam, Waltham, MA, USA) was also used. Slides were visualized using 3,3′-diaminobenzidine (DAB, K3468; Dako, USA). Stained slides were observed using the Scanscope AT scanner (Leica Biosystems, Wetzlar, Germany) and Vectra^®^ Automated Imaging System (version 2.6.0, Perkin Elmer Applied Biosystems, Waltham, MA, USA). Quantification of CD45, Aβ, and PD-1 expression levels were carried out using the Phenochart and Inform software available in the Vectra^®^ Automated Imaging System. Thioflavin-S staining was carried out by referring to previously reported methods [27]. Representative Thioflavin-S images were acquired using a confocal microscope (Zeiss LSM 780, Carl Zeiss AG, Jena, Germany).

Quantification of CD45, Aβ, and PD-1 expression levels were carried out using the Phenochart and In-form software available in the Vectra^®^ Automated Imaging System. Using the stamp tool available in the Phenochart (Vectra version 2.6.0) software, CD45-positive leukocyte infiltration was quantitated by drawing 2–4 rectangular ROIs (669 × 500 μm) in the left injected area. 10 ROIs (1338 × 1000 μm) were drawn on the left hemisphere to compare PD-1 expression differences between the 5xFAD hMSC and hMSC + DexaTofa groups. To quantitate % of amyloid burden (6E10 and Thioflavin-S), 5–7 ROIs (669 μm × 500 μm) were drawn on the left and right caudate putamen, respectively, of 5xFAD mice (MEM, hMSC, and DexaTofa). Sections posterior to the injected area were prepared to examine amyloid expression in the cortex, hippocampus, and thalamus of 5xFAD mice. For each brain region (cortex, hippocampus, and thalamus), 2 ROIs (1338 × 1000 μm) were drawn. The ROIs were drawn for both left and right hemispheres.

### 2.7. Whole-Transcriptome Sequencing Analysis

The left hemisphere (including the left cerebellum) of the harvested brain tissue samples was snap frozen in liquid nitrogen and subjected to RNA extraction and sequencing using the Illumina platform. Whole-Transcriptome Sequencing reads were aligned to the reference human genome (GRCh37, hg19) by STAR and the gene expression levels were quantified and determined based on Fragments per Kilobase per Million (FPKM) values. Log2-transformed FPKM values were employed for differentially expressed gene (DEG) analysis using DEseq2. To acquire the pathway level activities, we performed Gene Set Enrichment Analysis (GSEA) using the following parameters; Gene sets database: c2.all.v7.5.1.symbols, Number of permutations: 1000, Permutation type: gene_set.

### 2.8. Statistical Analysis

All values are expressed as mean ± standard error of mean (S.E.M). Statistical analyses were conducted using the GraphPad Prism 8.0 software (San Diego, CA, USA). One-way ANOVA (ordinary, multiple comparisons, Dunnett correction), two-way ANOVA (multiple comparisons, Tukey correction), or a *t*-test (unpaired, two-tailed, Welch’s correction) was used to assess significance. A *p*-value ≤ 0.5 was considered statistically significant.

## 3. Results

### 3.1. Combined Administration of Dexamethasone and Tofacitinib Demonstrated the Highest Quantity of Human Mesenchymal Stem Cells in the WT Mouse Parenchyma

**Figure 1:**
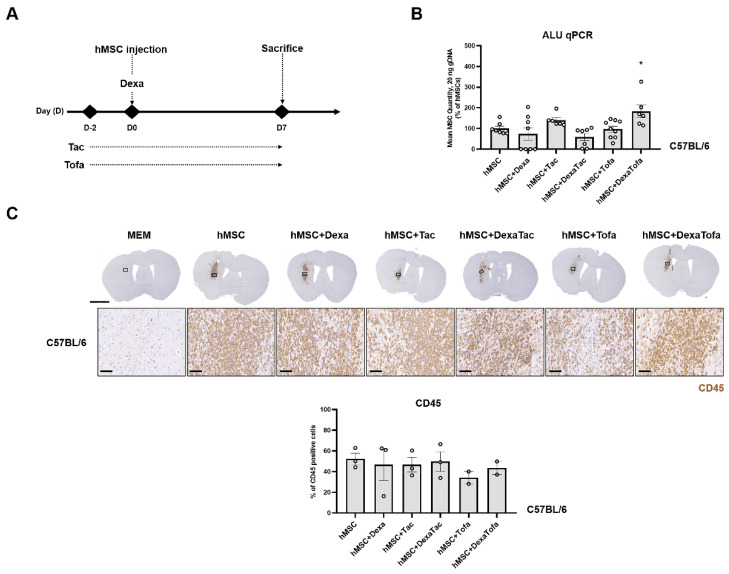
Examining the effects of various immunosuppressant regimens in WT mice transplanted with hMSCs. (**A**) Experimental Scheme. Dexamethasone (Dexa) was administered via the IV route at D0, right before and 4 h following hMSC transplantation. Both tacrolimus (Tac) and tofacitinib (Tofa) were administered orally starting at D-2 up to the sacrifice time point (D7). (**B**) Quantification of residual hMSCs in the WT mouse parenchyma carried out via ALU qPCR (hMSC: *n* = 7, hMSC+Dexa: *n* = 8, hMSC+Tac: *n* = 6, hMSC+DexaTac: *n* = 7, hMSC+Tofa: *n* = 9, hMSC+DexaTofa: *n* = 6). The highest number of residual hMSCs was observed from the hMSC + DexaTofa group. Statistical significance is defined as * *p* < 0.05 vs. hMSC; mean ± SEM (one-way ANOVA, Dunnett’s multiple comparison). (**C**) Representative images of CD45-positive leukocytes visualized as a dark brown precipitate (hMSC: *n* = 3, hMSC+Dexa: *n* = 3, hMSC+Tac: *n* = 3, hMSC+DexaTac: *n* = 3, hMSC+Tofa: *n* = 2, hMSC+DexaTofa: *n* = 2). Compared to hMSC transplantation alone, co-administration of immunosuppressants reduced leukocyte infiltration overall. Scale bars: whole brain =2 mm, magnified inset area (solid black box) = 60 µm.

Prior to administering immunosuppressants in 5xFAD mice, administrations were first performed using WT mice to narrow down the immunosuppressant regimen condition. As shown in Figure 1A, dexamethasone (Dexa) was administered on the day of cell transplantation while tacrolimus (Tac) and tofacitinib (Tofa) were administered starting at D-2 (2 days before the cell transplantation). A total of five different immunosuppressant regimen conditions were assessed using WT mice (hMSC + Dexa, hMSC + Tac, hMSC + DexaTac, hMSC + Tofa, and hMSC + DexaTofa). Compared to the hMSC group, a higher number of residual hMSCs was identified from the hMSC + Tac and hMSC + DexaTofa groups (Figure 1B). A statistically significant difference was identified from the hMSC + DexaTofa group and compared to the hMSC group, the number of residual hMSCs increased by approximately 1.8-fold for the hMSC + DexaTofa group (* *p* = 0.0444, one-way ANOVA, Dunnett’s multiple comparison, hMSC = control). Compared to the hMSC group, an increase in the quantity of residual hMSCs was not noted from the hMSC + Dexa, hMSC + DexaTac, and hMSC + Tofa groups. To examine the effects of the varying immunosuppressant regimens on the immune response exerted by the transplanted hMSCs, immunohistochemical (IHC) analysis was performed. As reported previously [3,4], a dense population of CD45-positive leukocytes, visible as DAB positive-dark brown precipitates, infiltrated the area where hMSCs were transplanted (Figure 1C). Other than damage to the cortex which may have occurred via penetration of the Hamilton syringe into the parenchyma, infiltration of CD45-positive leukocytes was not identified from mice injected with MEM media only. Overall, a decrease in the infiltration of leukocytes was noted in all groups that received administrations of immunosuppressants (Compared to the hMSC group, the hMSC + Tofa group showed the highest reduction in infiltration of leukocytes followed by the hMSC + DexaTofa group. While a statistically significant difference was not noted when comparing the hMSC + DexaTofa group to the hMSC group (*p* = 0.9571; one-way ANOVA, Dunnett’s multiple comparisons test, hMSC = control), overall, the hMSC + DexaTofa group was effective in increasing hMSC persistence and decreasing immune responses generated by the injected hMSCs. Thus, the dexamethasone + tofacitinib condition was chosen out of the various immunosuppressant regimens to be applied to the 5xFAD mouse model.

### 3.2. Downregulation of Immune Responses following Combined Administration of Dexamethasone and Tofacitinib in 5xFAD Mice

**Figure 2:**
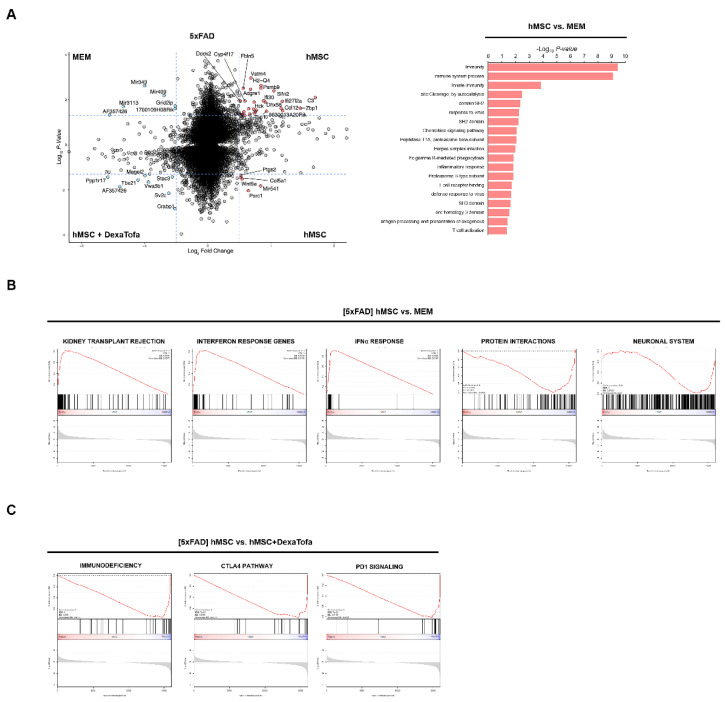
Whole-Transcriptome sequencing analysis revealed that combined administration of dexamethasone and tofacitinib reduced immune response in 5xFAD mice. (**A**) Differentially Expressed Genes (DEGs) between hMSC vs. MEM and hMSC vs. hMSC + DexaTofa. Red nodes represent genes that are upregulated in the hMSC (*n* = 2) group, while blue nodes represent genes upregulated by the MEM (top panel, *n* = 2) or hMSC + DexaTofa (bottom panel, *n* = 2) groups. Gene Ontology (GO) analysis of DEGs (right panel). (**B**) Gene Set Enrichment Analysis (GSEA) indicated that compared to MEM media administration, transplantation of hMSCs demonstrated enrichments of gene sets associated with immune response. (**C**) When compared to the hMSC group via GSEA, gene sets associated with immunodeficiency are revealed from the hMSC + DexaTofa group.

To elucidate the underlying transcriptional signatures or pathways involved in the immunological response to human mesenchymal stem cell transplantation, we compared the transcriptome profiles of 5xFAD mice injected with either MEM media or hMSC. Notably, Differentially Expressed Gene (DEG) analysis demonstrated increased transcriptional levels of Adgre1, Hck, and Dhx58, primarily involved in pathways that are associated with immune response, inflammation, and chemokine signalling pathway in the hMSC group (Figure 2A,B). Consistently, Gene Set Enrichment Analysis (GSEA) exhibited activation of gene-sets that were related to IFN¦Á response, kidney transplant rejection, and interferon response in the hMSC group, whereas the MEM group showed increased activities of protein interactions and neuronal system (Figure 2B). When we compared the gene expression profiles of the hMSC group compared to the hMSC + DexaTofa group, we discovered enrichments of pathways that were involved in T cell exhaustion, including immunodeficiency, CTLA4, and PD1 signalling pathways (Figure 2C). Collectively, whole-transcriptome analysis revealed profound levels of immunological changes in response to hMSC transplantation and immunosuppression treatment, specifically in interferon-mediated T cell activities.

### 3.3. Transcriptome Sequencing Results Were Corroborated via Immunohistochemical Analysis

**Figure 3:**
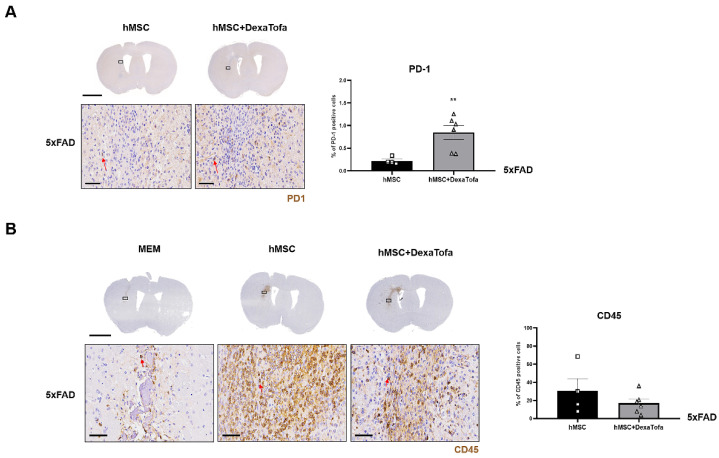
Combined administrations of dexamethasone and tofacitinib reduced PD-1 expression. (**A**) Representative images of PD-1 positive cells (solid red arrows) in the left hemisphere of hMSC (*n* = 4) and hMSC + DexaTofa (*n* = 6) groups. Higher PD-1 expression is evident from the hMSC + DexaTofa group. Statistical significance was defined as ** *p* < 0.01 vs. hMSC; mean ± SEM (unpaired t-test). Scale bars: whole brain = 2 mm, magnified inset area (solid black box) = 60 µm. (**B**) Representative images of CD45-positive leukocytes (dark brown precipitate, solid red arrows) for the MEM (*n* = 3), hMSC (*n* = 4), and hMSC + DexaTofa (*n* = 6) groups. Compared to the hMSC group, the hMSC + DexaTofa group displayed reduced levels of CD45-positive leukocytes. Scale bars: whole brain = 2 mm, magnified inset area = 60 µm. Values are expressed as mean ± SEM.

To corroborate the RNA sequencing results, the expression of PD-1 in the injected area (left hemisphere) was evaluated via IHC (Figure 3A). The PD-1 positive cells were identified as cells with dark brown precipitates surrounding the nuclei (red arrow). A higher number of PD-1 positive cells were identified in the 5xFAD hMSC + DexaTofa group in comparison to that of the 5xFAD hMSC group. The difference was also statistically significant (** *p* = 0.0086; unpaired *t*-test with Welch’s correction). Like WT mice, when tissue sections obtained from 5xFAD mice were stained with the CD45 antibody, a high infiltration of CD45-positive leukocytes (dark brown precipitate) was identified from the hMSC group (Figure 3B). Reduced infiltration of CD45-positive leukocytes from the 5xFAD hMSC + DexaTofa group. While damage to the parenchyma was identified in the MEM group due to the insertion of the Hamilton syringe, profound levels of leukocyte infiltration were not exhibited when compared to that of the hMSC and hMSC + DexaTofa groups. Differences between the two groups, however, was not statistically significant (*p* = 0.3958; unpaired *t*-test with Welch’s correction).

### 3.4. Combined Administration of Dexamethasone and Tofacitinib Increased hMSC Persistence in the Parenchyma of 5xFAD Mice

**Figure 4:**
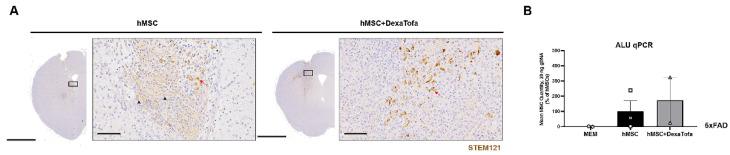
Quantification of residual hMSCs in the 5xFAD parenchyma. (**A**). Representative images of STEM121 positive cells (residual hMSCs). Non-specific signals are present (solid black arrowhead). Residual hMSCs (solid red arrows) were barely detected at D7 for both the hMSC and hMSC + DexaTofa groups. Scale bars: whole brain = 2 mm, magnified inset area (solid black box) = 60 µm. (**B**) ALU-qPCR was used to quantify the number of residual hMSCs present in the parenchyma for both the hMSC (*n* = 3) and hMSC + DexaTofa (*n* = 2) groups. Human DNA was not detected from the MEM (*n* = 2) group and compared to the hMSC group, higher MSC quantity was revealed (difference is not statistically significant) from the hMSC + DexaTofa group. Values are expressed as mean ± SEM.

To identify the presence of hMSCs in the 5xFAD parenchyma, IHC was carried out using the STEM121 antibody (Figure 4). In our previously reported studies [3,4] (compared to the 0 h group (mice sacrificed immediately following cell transplantation), residual hMSCs were barely identified from the hMSC group that was sacrificed a week following transplantation. Similar to our past reports, very few STEM121 positive cells (dark brown precipitate, red arrowhead) were detected from both the 5xFAD hMSC and hMSC + DexaTofa groups. The presence of non-specific signals (solid black arrow) also made it more difficult to exactly pinpoint the presence of residual hMSCs in the mouse parenchyma. Therefore, ALU qPCR was used to quantify the number of residual hMSCs in the 5xFAD parenchyma (left hemisphere). Like the PCR results observed from WT mice, an increase in number of residual hMSCs was observed from the hMSC + DexaTofa group in comparison to that of the hMSC group (Figure 4B). The difference, however, was not statistically significant (*p* = 0.7074; unpaired *t*-test with Welch’s correction).

### 3.5. Significant Reduction of Amyloid Burden Was Not Evident in the Injected Area of 5xFAD Mice

**Figure 5:**
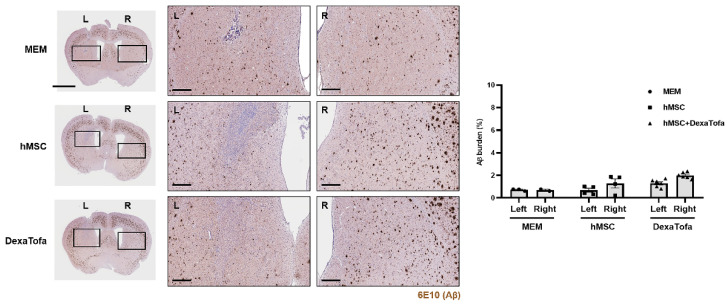
Comparison of amyloid burden between the injected (left caudate putamen) and non-injected (right caudate putamen) areas of 5xFAD mice. Representative images of 6E10 stained sections to assess amyloid deposition in the left (injected area) and right caudate putamen of the MEM (*n* = 3), hMSC (*n* = 4), and hMSC + DexaTofa groups (*n* = 6). L: left, R: right. Beta amyloid was visualized as dark brown precipitates. The injected area (left putamen) displayed reduced levels of amyloid deposition for both the hMSC and hMSC + DexaTofa groups. but the differences between the two hemispheres were statistically insignificant (two-way ANOVA, Tukey’s multiple comparison). Scale bars: whole brain = 2 mm, magnified inset area = 300 µm. Values are expressed as mean ± SEM.

To examine the combined effects of hMSC injection and DexaTofa administration on amyloid deposition, IHC was performed using the 6E10-Aβ antibody (Figure 5). Amyloid burden (dark brown precipitates) of the injected area (left caudate putamen) was compared to that of the opposite caudate putamen (right). Compared to the right caudate putamen, a slight reduction in amyloid burden was exhibited in the injected area (left) of the hMSC group but the difference was not statistically significant (two-way ANOVA, Tukey’s multiple comparison, *p* = 0.3103, F = 1.432, DFn = 2, DFd = 19). Amyloid deposition was greater by approximately 1.9-fold in the right caudate putamen. For the hMSC + DexaTofa group, the left injected area displayed reduced levels of amyloid burden compared to that of the right caudate putamen hMSC group; the difference between the two hemispheres was not statistically significant (two-way ANOVA, Tukey’s multiple comparison, *p* = 0.0709, F = 1.43, DFn = 2, DFd = 19). Amyloid deposition was greater by approximately 1.6-fold in the right caudate putamen. Contrary to our expectation, amyloid levels, overall, were higher for the hMSC and hMSC + DexaTofa groups in comparison to that of the MEM group.

**Figure 6:**
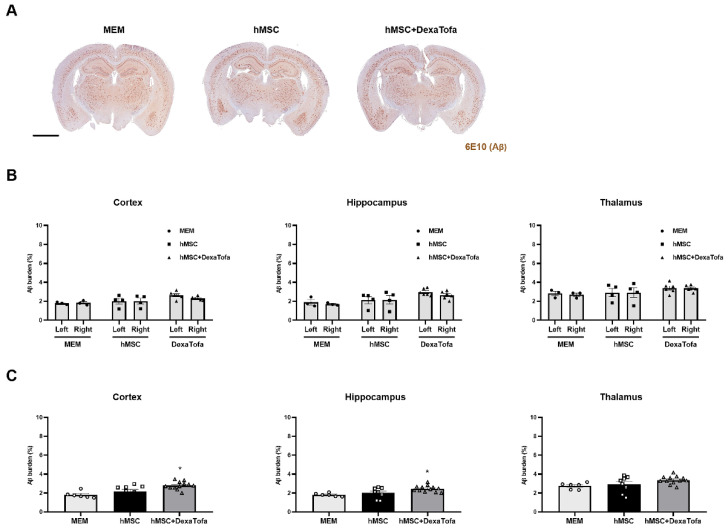
Assessment of changes in amyloid deposition in the cortex, hippocampus, and thalamus. (**A**) Representative 5xFAD brain tissue sections acquired from the following groups: MEM (*n* = 3), hMSC, and hMSC (*n* = 4) + DexaTofa (*n* = 6) were stained using the 6E10 antibody to examine differences in amyloid burden (dark brown precipitate) among the groups. Scale bar: whole brain = 2 mm. (**B**) The amyloid burden of the left and right hemispheres was evaluated first in the following three regions: hippocampus, and thalamus. Significant differences between the hemispheres were not noted for all three regions. mean ± SEM (two-way ANOVA, Tukey’s multiple comparison). (**C**) Data acquired from the left and right hemispheres were combined for each of the three regions. Out of the three groups, the hMSC + DexaTofa group displayed the highest level of amyloid deposition for all three regions. Statistical significance is defined as * *p* < 0.05 vs. hMSC; mean ± SEM (one-way ANOVA, Dunnett’s multiple comparison).

In addition to the analysis for the injected area, we performed the same analysis for three different regions: cortex, hippocampus, and thalamus (Figure 6A). Compared to the left hemisphere, a statistically significant difference (two-way ANOVA, Tukey’s multiple comparison) in amyloid deposition was not detected in the following brain regions of the right hemisphere for the MEM, hMSC, and hMSC+DexaTofa groups (F = 0.2402, DFn = 2, DFd = 20, hippocampus (F = 0.5861, DFn = 2, DFd = 20), thalamus (F = 0.02239, DFn = 2, DFd = 20) (Figure 6B). Data from the left and right hemispheres were also combined and analysed (Figure 6C). Similar to the assessment of amyloid deposition in the caudate putamen, the hMSC and hMSC + DexaTofa groups displayed higher amyloid levels in comparison to that of the MEM group. Moreover, amyloid burden of the hMSC + DexaTofa group was statistically significant when compared to that of the hMSC group (one-way ANOVA, Dunnett’s multiple comparison, hMSC = control) in both the cortex (* *p* = 0.0230) and hippocampus (* *p* = 0.0416). A statistically significant difference between the two groups was not noted in the thalamus (*p* = 0.1652).

**Figure 7:**
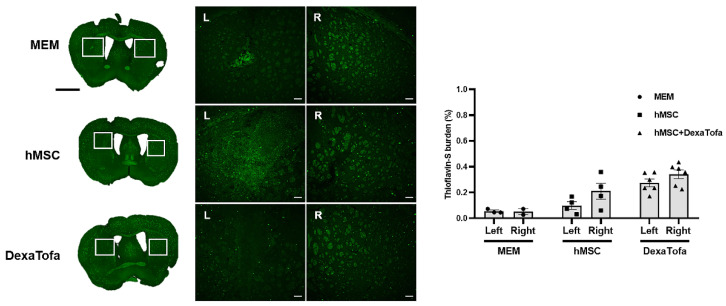
Thioflavin-S staining of the left and right caudate putamen. Representative Thioflavin-S-stained tissue images of the MEM (*n* = 3), hMSC (*n* = 4), and DexaTofa (*n* = 6) groups acquired via Vectra (whole brain) and confocal microscopy (magnified image). Green dots (fluorescence signal) indicate location of amyloid plaques. Scale bars = whole brain: 2 mm, magnified inset area = 100 µm. (L vs. R) mean ± SEM (two-way ANOVA, Tukey’s multiple comparison).

**Figure 8:**
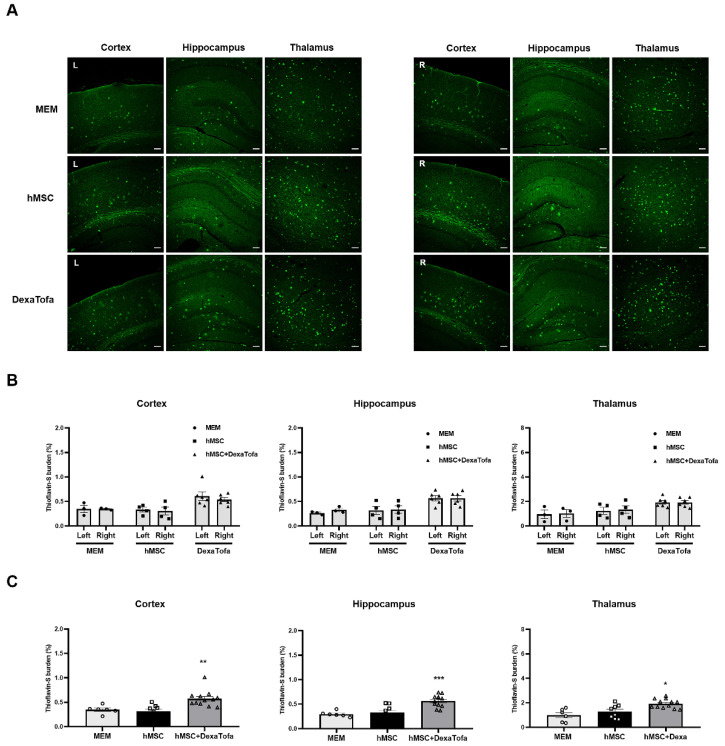
Thioflavin-S staining of the cortex, hippocampus, and thalamus of 5xFAD mice. (**A**) Representative Thioflavin-S-stained tissue images of the MEM (*n* = 3), hMSC (*n* = 4), and hMSC+DexaTofa (*n* = 6) groups were acquired via a confocal microscope. Green dots (fluorescence signal) indicate the location of amyloid plaques. Scale bar for each image = 100 µm. (**B**) Thioflavin-S burden % of the left (L) and right (R) hemispheres were quantitated for the cortex, hippocampus, and thalamus. Differences between the left and right hemispheres were not statistically significant for all 3 regions (mean ± SEM, two-way ANOVA, Tukey’s multiple comparison). (**C**) Data collected from the left and right hemispheres were combined and averaged for each region Statistical significance is defined as * *p* < 0.05, ** *p* < 0.01, *** *p* < 0.001 vs. hMSC; mean ± SEM (one-way ANOVA, Dunnett’s multiple comparison).

Thioflavin-S staining was also carried out to examine amyloid deposition in the 5xFAD parenchyma. According to the images acquired via a confocal microscope, overall, very few amyloid plaques (fluorescence signal identified as green dots) were identified in the caudate putamen of all three groups (MEM, hMSC, and DexaTofa) (Figure 7). However, like the IHC results obtained using the 6E10 antibody, compared to the right caudate putamen, a slight reduction in Thioflavin-S burden (%) was identified in the left caudate putamen of the hMSC (*p* = 0.4006) and DexaTofa (*p* = 0.6945) groups. The difference between the left and right caudate putamen of the hMSC and DexaTofa groups, respectively, was not statistically significant (two-way ANOVA, Tukey’s multiple comparison, F = 0.7781, DFn = 2, DFd = 19). Statistically significant results were also not observed when the left and right hemispheres of the hMSC and hMSC + DexaTofa groups were analysed for the cortex (F = 0.1290, DFn = 2, DFd = 20), hippocampus (F = 0.1396, DFn = 2, DFd = 20), and thalamus (F = 0.02432, DFn = 2, Dfd = 20) (Figure 8A,B). However, when the results of the left and right hemispheres were combined (Figure 8C), compared to the hMSC group (one-way ANOVA, Dunnett’s multiple comparison, hMSC = control), the Thioflavin-S burden % was higher for the DexaTofa group in the cortex (** *p* = 0.0010), hippocampus (*** *p* = 0.0006), and thalamus (** *p* = 0.0121).

## 4. Discussion

The results of this study support the combined application of immunosuppressants and MSCs for AD stem cell therapy. As hypothesized, an immunosuppressant regimen involving the use of more than one immunosuppressant (dexamethasone and tofacitnib combined; DexaTofa) was effective in reducing immune responses generated via injection of xenogeneic human MSCs into the caudate putamen of 5xFAD mice. To date, very few studies have investigated the use of more than one immunosuppressant, let alone a single immunosuppressant, in modulating immune responses exerted via hMSCs transplanted into the parenchyma of a transgenic AD mouse model. Many groups, however, have reported on how administration of a single immunosuppressant (without cell transplantation) can inhibit immune responses and or augment therapeutic benefits in various mouse models. Although not used in this study, administration of an immunosuppressant called rapamycin ameliorated the AD pathology and improved the cognitive performance of a transgenic AD mouse model [28].

Dexamethasone is a glucocorticoid that has been widely used clinically as a treatment regimen for a variety of diseases including infectious diseases of the central nervous system (CNS) [29]. Glucocorticoids are known to affect multiple types of immune cells [30] and to be effective in suppressing T-cell proliferation [31]. One study reported that when peripheral blood natural killer (NK) cells were treated with 10 and 100 ng/mL of dexamethasone in-vitro, dexamethasone significantly inhibited the activation of natural killer (NK) cells [32]. When co-cultured with MSCs, dexamethasone did not inhibit but more so augmented the inhibitory effects of MSCs on NK cell activation [32]. A clinical trial used a 2.5 mg of dexamethasone that was administered intravenously prior to transplantation of allogeneic umbilical cord (UC)-MSCs in type 1 diabetes patients [33]. Previous cases have reported on how long-term use (several months) of corticosteroids may cause detrimental effects [34]. However, these adverse effects were reversible. Because a short-term use of dexamethasone (recommended doses) will be incorporated into stem cell therapy, this will play a significant role in minimizing side effects. Corticosteroids are also known to reduce fever [35], which may serve beneficial purposes in AD stem cell therapy considering that transient fever was previously observed in a phase I clinical trial within 24 h after injecting human umbilical cord blood-derived MSCs via the intracerebroventricular route in AD patients [36]. Like all immunosuppressants, dosage dependent adverse effects may arise, but the injected dose will be diluted upon administration and the mean plasma concentration of dexamethasone will decrease over time [37]. The dosage used in this current study has been used clinically. Although further study is warranted, due to the diluted effects of dexamethasone, the injected dose is less likely to cause adverse effects on the preservation or engraftment of the transplanted stem cells but ameliorate immune/inflammatory responses generated by the transplanted MSCs.

Although commonly used for the treatment of rheumatoid arthritis [38], the potential efficacy of tofacitinib, a Janus kinase (JAK) 1 and 3 inhibitor, in CNS autoimmune diseases has recently been reported [39]. The anti-inflammatory effects of tofacitinib include inhibition of T-cell proliferation and suppression of innate immune cell function [40]. One group reported that tofacitinib was effective in suppressing macrophage-mediated xenogeneic cytotoxicity [41]. This attribute of tofacitinib is essential in that macrophage infiltration is a direct effector of xenograft rejection [42]. Contrary to dexamethasone, the combined use of tofacitinib and mesenchymal stem cells has not been extensively studied in both in-vitro and in-vivo levels.

The upregulation of immune-related differentially expressed genes in 5xFAD mice transplanted with hMSCs add to the growing evidence that MSCs may not be hypoimmunogenic. For years it has been widely accepted that MSCs are not immunogenic because they first, express low levels of the major histocompatibility complex (MHC) class I and second, do not express MHC class II [43]. The expression of MHC molecules seems to play a vital role in evading the recipient’s immune recognition [44]. The hMSCs used in this research have been characterized as previously reported [24] and via fluorescence activated cell sorting (FACS), we have confirmed that the cells do not express human leukocyte antigen (HLA)-DR (HLA class II). Thus, it is questionable as to why hMSCs with these immunophenotypic characteristics mediated immune responses upon transplantation into the mouse brain. One group has published findings that HLA class II may be upregulated during the in-vitro expansion of MSCs and that such changes will generate immunological reactions after the MSCs are injected in-vivo [45,46,47]. Another study has proposed that changes in MHC class I expression may occur following transplantation and that such phenotypic changes can impact cell transplant outcomes [48]. Although not examined in this study, we cannot rule out the possibility that the hypoimmunogenic properties of the hMSCs may have been compromised following intraparenchymal administration.

Our data suggest that the combined use of the immunosuppressants, dexamethasone and tofacitinib (DexaTofa), in 5xFAD mice transplanted with hMSCs, was effective in upregulating pathways associated with immunodeficiency and PD-1 signalling. An increase in expression of regulatory T cells (Tregs) signifies suppression of immune response [49]. The PD-1/PDL1 signalling pathway serves as an immune checkpoint and allows tumour cells to evade immune recognition from the host [50]. The interaction that occurs in between PD-1 of immune cells and PDL-1 of tumour cells allows tumour cells to escape the attack of immune cells such as T-cells. Transcriptome sequencing results were corroborated via immunohistochemical staining where a significant increase in PD-1 expression was displayed in the injected area (left hemisphere) of the hMSC + DexaTofa group in comparison to that of the hMSC group. These results are indicative that the combined use of dexamethasone and tofacitinib was effective in moderating the immune system of 5xFAD mice transplanted with hMSCs.

As we have reported previously [3], compared to the 0 h group (sacrificed right after MSC transplantation) where STEM121 positive signals were clearly identified, it was difficult to pinpoint the exact location of the transplanted hMSCs in the D7 group because very few persisting hMSCs were identified in the mouse parenchyma. Furthermore, non-specific signals were also identified which hindered the clear discrimination between positive and false signals. Apoptotic and necrotic cells possibly present at the injection area [3] along with infiltration of immune cells may have generated these non-specific signals. Thus, the effects of DexaTofa on MSC graft survival was assessed directly via real time PCR where residual hMSCs in the mouse parenchyma were quantified. Compared to the 5xFAD hMSC group, a higher number of residual hMSCs was detected from the 5xFAD hMSC + DexaTofa group. Such results were similar to that observed from WT mice.

Our findings are in concordance with previous studies where very few amyloid plaques were discernible in the striatum [51] of 5xFAD mice, particularly at the age of 9 to 10 months. Compared to Thioflavin-S staining, amyloid expression was overall higher for all three groups (MEM, hMSC, and hMSC+DexaTofa) when IHC was carried out using the 6E10 antibody. This difference can be attributed to sensitivity in detecting amyloid deposition [52,53]. The 6E10 antibody, for example, is known to cross react with the amyloid precursor protein (APP) or APP c-terminal fragments [53,54]. The antibody has also been reported to be expressed by immune/inflammatory cells [55]. Such attributes of the 6E10 antibody should be taken into consideration for future experiments where along with 6E10 a confirmatory experiment (IHC) should be conducted using at least one additional amyloid beta marker.

Unexpectedly, compared to the MEM injected group, higher amyloid deposition was exhibited from the hMSC + DexaTofa group. Such results could have arisen due to the individual variability of the mice for each group, small sample size, or early sacrifice time point (post 1 week). We also cannot rule out differences in gender. An equal number of male and female 5xFAD mice were not used to carry out histological analysis in this study. Previously, a group has reported that female 5xFAD mice show higher levels of human APP and amyloid-β than the male counterparts [56].

The sacrifice time point (1 week following cell transplantation) may have been relatively too short to observe significant amyloid reduction in the hMSC and hMSC + DexaTofa groups. Although not examined in this study, it is possible that amelioration of amyloid burden may have been identified if mice were sacrificed at an extended time point. For example, reduction of amyloid plaques and decrease in both soluble and insoluble Aβ40 and Aβ42 levels were demonstrated from APP/PS1 mice sacrificed 40 days after receiving intraparenchymal injections of human umbilical cord-blood derived mesenchymal stem cells [57]. Improvement in cognitive performance along with reduction in Aβ42 density levels in the fornix and subiculum of 5xFAD mice were noted 10 weeks after carrying out intraparenchymal injections of bone marrow derived allogeneic mouse MSCs [15].

This study has several limitations. The first limitation is sample size. Results acquired using WT mice (Figure 1) were referred to narrow down the immunosuppressant regimen for 5xFAD mice. However, due to the small sample size, we were not able to observe statistical significance when comparing the hMSC + immunosuppressant groups to the hMSC only group in relation to CD45 leukocyte infiltration at the injected area. Due to the exclusion of two mice, the sample size of the 5xFAD hMSC + DexaTofa group ALU qPCR -based quantification was too small to achieve statistical significance. The second limitation is that varying doses and administration routes of dexamethasone and tofacitinib were not evaluated in the hMSC-transplanted 5xFAD mice. One of the objectives of the study was to apply clinically relevant doses and administration routes of the immunosuppressants. Thus, the effects of how different doses and administration routes of dexamethasone and tofacitinib may alter MSC engraftment and MSC-exerted immune responses have not been comprehensively examined in this study. The third limitation is the use of a single transgenic AD mouse model. Transgenic AD mouse models available for research are not able to recapitulate the entire, complicated pathology of Alzheimer’s disease. Thus, to facilitate the use of immunosuppressants in AD stem cell therapy, the efficacy of the selected immunosuppressant regimen should be tested in multiple AD mouse models.

## Figures and Tables

**Table 1:** Route of Administration and Dosing Schedules for Immunosuppressants.

Immunosuppressant	Route of Administration	Dosing Schedule	Clinical Dose
Dexamethasone (Dexa)	IV	2.1 mg/kg, (0 h and 4 h) ^1^	10 mg/60 kg
Tacrolimus (Tac)	P.O.	0.6 mg/kg, qd ^2^	3 mg/60 kg
Tofacitinib (Tofa)	P.O.	1 mg/kg, bid ^3^	5 mg/60 kg

^1^ Administered at D0 (day of cell injection). ^2^ Administered daily from D-2 to D7. ^3^ Administered twice a day.

## Data Availability

The data presented in this study are available on request from the corresponding author.
